# Segmentation of MRI Brain Images with an Improved Harmony Searching Algorithm

**DOI:** 10.1155/2016/4516376

**Published:** 2016-06-15

**Authors:** Zhang Yang, Ye Shufan, Guo Li, Ding Weifeng

**Affiliations:** ^1^School of Information and Engineering, Wenzhou Medical University, Wenzhou, Zhejiang 325000, China; ^2^Zhejiang ZhongLan Environment Technology Ltd., Wenzhou, Zhejiang 325000, China; ^3^School of Medical Imaging, Tianjin Medical University, Wenzhou, Zhejiang 300000, China; ^4^118 Hospital of the People's Liberation Army, Wenzhou, Zhejiang 325000, China

## Abstract

The harmony searching (HS) algorithm is a kind of optimization search algorithm currently applied in many practical problems. The HS algorithm constantly revises variables in the harmony database and the probability of different values that can be used to complete iteration convergence to achieve the optimal effect. Accordingly, this study proposed a modified algorithm to improve the efficiency of the algorithm. First, a rough set algorithm was employed to improve the convergence and accuracy of the HS algorithm. Then, the optimal value was obtained using the improved HS algorithm. The optimal value of convergence was employed as the initial value of the fuzzy clustering algorithm for segmenting magnetic resonance imaging (MRI) brain images. Experimental results showed that the improved HS algorithm attained better convergence and more accurate results than those of the original HS algorithm. In our study, the MRI image segmentation effect of the improved algorithm was superior to that of the original fuzzy clustering method.

## 1. Introduction

With the development of image processing technology, medical imaging technology has significantly improved. A wide variety of medical images are currently being produced. Several currently available imaging approaches include computed tomography, magnetic resonance imaging (MRI), and ultrasound. These techniques are extensively used in medical diagnosis, preoperative planning, treatment, and postmanagement detection. MRI is commonly used in actual clinical diagnosis. Compared with other technologies, MRI does not employ radiation on the human body. At the same time, high-resolution imaging of human soft tissue is attained, which can be achieved in any Italian dimensional imaging [1–4]. Although MRI technology is extensively used in medicine, MRI data and images can be generated under objective and subjective reasons for data transmission as well as the environment, and other instruments produce gradation unevenness and offset field effect. Limited resolutions produce similar noise effects. Therefore, improving MRI technology is important to enhance analysis. Under MRI, the skull is relatively bright white. The range of gray values in the skull and white matter usually overlaps. The skull bone and muscle exhibit gray values similar to those of brain tissue. In a segment containing white and gray matter, the skull is resolved together with the white matter. Therefore, the accurate segmentation of MRI images is important to eliminate interference. Methods for regional enlargement is suitable for achieving clear image segmentation of the target boundaries. If the target is unclear, then the image cannot be effectively extracted. Dynamic contour models generate enhanced segmentation effects but are disadvantageous because of long computing time. Meanwhile, deformable model methods are divided into two categories, namely, the parametric deformable model and variable-level set-shape model. These methods are achieved by iterative calculation, which takes a long time. Manual determination of iteration points must first be achieved. Given the involvement of personal and subjective factors, the segmentation attained by such method is unstable. Mathematical morphological imaging of the skull for removal treatment is effective, but a suitable threshold is more difficult to achieve using such technique. Falcao et al. [5] proposed the use of a live-wire segmentation algorithm. The algorithm can provide the user effective control of the segmentation process. In the approach, the user can intervene with the results of segmentation. Another method utilizes the artificial neural network (ANN), which is composed of many processing units (nodes). The ANN can simulate the biological, particularly the massively parallel, network of the human brain learning process. Input data acquire results quickly by training under ANN theory. With such strategy, the speed of image segmentation is effectively improved. The neural network algorithm does not entail prior knowledge of the probability distribution of image gray values; consequently, segmentation results are similar to the original image [6]. The neural network method shows its unique advantages in solving a series of complex image segmentations; however, several issues arise. First, the energy function in such case falls into local minimum values for minimized images. Second, the convergence of the neural network is related to the data; thus, a suitable value for testing network inputs is needed. The fuzzy clustering method is more extensively applied for image segmentation. The fuzzy clustering algorithm has the following advantages: the algorithm avoids the issue of threshold setting and does not entail human manipulation. Furthermore, the fuzzy clustering algorithm is particularly suitable for fuzzy and uncertain images. In this study, the fuzzy *c*-means clustering algorithm was selected to segment MRI brain images. Scholars discovered that the effect of the initial value of the cluster centers of the fuzzy clustering algorithm is relatively large. The characteristics of a nonconvex function involve several local minima; thus, the initial value of FCM will fall into the local minima. This study utilized the rough set to compute for the initial value of the FCM.

The harmony searching (HS) algorithm was developed by Korean scholars. Geem et al. [7, 8] proposed a kind of intelligent optimization algorithm in 2001. The algorithm describes the process of musical improvisation; different musical tones are applied to a harmony vector to search for a harmony randomly. Then, the process attains an optimal harmony. Jang et al. [9] used the Nelder-Mead simplex HS algorithm, whereas Mahdavi et al. [10] adopted the adaptive HS algorithm (IHS). Omran and Mahdavi compared the performances, parameters, and noise effects related to the original HS algorithm [11], IHS, and global-best HS algorithm. H.-Q. Li and L. Li [12] employed the genetic algorithm and the HS algorithm to explore the three functions of Rastrigin, Griewank, and Sphere. Liang and coworkers [13, 14] adjusted certain parameters to improve the HS algorithm and used a hybrid GA-HS algorithm to solve the critical sliding slope problem. Cheng et al. [15] adopted the HS algorithm with several other heuristic optimization algorithms for earth slope stability analysis. Dong et al. [16] proposed the HS *k*-means clustering algorithm to change WEB text categorization. Bezdek [17] utilized an adaptive adjustment of parameters on the improved HS algorithm to solve the anomaly detection problems in digital images of biological tissue.

In recent years, the HS algorithm has been adopted in several applications. However, the algorithm exhibits several disadvantages. The HS algorithm operates with weak robustness, considerable randomness, lack of specific direction, and slow convergence speed; it easily falls into the local optimal solution. The problem can be attributed to the search mechanism of the HS algorithm. This study proposed an improved HS algorithm for MRI brain image segmentation to overcome the aforementioned disadvantages. We used the rough set and memory bank of the HS algorithm together with the concept of rough set upper and lower boundary correction HS algorithm of the “optimal” and “worst” harmonies. By doing so, we prevented the HS algorithm convergences from falling into the local optimum. The HS algorithm should be employed to obtain the number of optimal solutions as the initial value for the average fuzzy clustering algorithm. This strategy would overcome the random determination of the initial value of the fuzzy clustering algorithm. Experimental results showed that the proposed algorithm achieved perfect convergence, and the segmentation effect was ideal for the MRI brain images. Besides ANN and fuzzy clustering, ensemble learning [18, 19], feature ranking [20], and samples selection [21] were also employed in the biomedical research.

## 2. Harmony Searching Algorithm

The HS algorithm has been proposed as a new algorithm for the study of musical play. Each musician produces individual tones that can generate vector values. If the music produced is pleasant sounding, then the tone is recorded and tools are employed to generate a better harmony in the subsequent attempt. Musical harmony is analogous to the optimal solution vector, whereas the player riffs correspond to the optimization techniques in the local and global search programs. The HS algorithm uses a random search that selects probabilities and adjusts the pitch without information derived from the harmony. Compared with early heuristic optimization algorithms, the HS algorithm is conceptually straightforward, utilizing less mathematical expressions and a few parameters for the random search of theoretical values. Moreover, the algorithm can be more easily optimized for various engineering problems. These theoretical ideas can be adopted to formulate the solution vector *X* ^*j*^ = (*X*_1_^*j*^, *X*_2_^*j*^,…, *X*_*n*_^*j*^), which refers to the evaluation function for *f*(*X* ^*j*^). The HS algorithm is mainly divided into the following steps.

**Step 1 (initialization parameter).**

The HS algorithm includes a series of important parameters, such as the number of iterations *k*; the harmony memory data base HM; the harmony memory probability values PAR_max_ and PAR_min_; the fine-tuning probability BW; the harmony memory size HMS; the dimension parameter optimization problem *N*; and the upper and lower boundaries *x* ^U^ and *x* ^L^, respectively.

**Step 2 (harmony memory initialization).**

A harmony database is adopted to store the HMS of random harmonic vectors. The random harmonic vectors by weight of each dimension on the upper and lower boundaries *x* ^U^ and *x* ^L^, respectively, are expressed as follows:

(1) $$\begin{array}{c} x_{i}=x^{L}+\mathrm{rand}\left(\;x^{U}-x^{L}\;\right)\;i=1,2,\ldots N. \end{array}$$

The HM matrix expression is as follows:

(2) $$\begin{array}{c} HM=\left|\begin{array}{cccc} x_{1}^{1} & x_{2}^{1} & \cdots & x_{N}^{1}\;|\;f\left(\vec{x^{1}}\right)\\ x_{1}^{2} & x_{2}^{2} & \cdots & x_{N}^{1}\;|\;f\left(\vec{x^{2}}\right)\\ \vdots & \vdots & \vdots & \vdots \\ x_{1}^{HMS} & x_{1}^{HMS} & \cdots & x_{1}^{HMS}\;|\;f\left(\vec{x^{HMS}}\right) \end{array}\right|. \end{array}$$

**Step 3 (new harmony generation).**

In accordance with the change in objective function value, the adaptive setting of the harmony memory considers the probability HMCR. By the maximum and minimum sounds of the initial setup, probability dynamic adjustment PAR is achieved. After parameter adjustment, learning by the differences in operation, pitch adjustment, and random mutation process, new harmonic solution vectors are created.

The process involved is as follows:

 
*For i* ∈ [1, *N*]  %* Beginning,*
 
*if *rand⁡( ) < = HMCR* then*
   
*x*_*i*_′ = *x*_*i*_/(*j* = *ceil*(rand⁡( )*∗*HMS))  %* memory consideration*
   
*if *rand⁡( ) < = PAR* then*
   
*x*_*i*_′ = *x*_*i*_ ± rand⁡( )*∗*BW  %* pitch adjustment*
 
*else*
   
*x*_*i*_′ = *x*_*i*_^L^ + rand⁡( )*∗*(*x*_*i*_^L^ − *x*_*i*_^U^)  %* random selection*
 
*end if*

**Step 4 (memory bank updating).**

The new harmony is regarded as the worst harmony in the database, and the most optimal update for the worst harmony is utilized in the database.

**Step 5 (termination conditions).**

The current number of iterations is determined to achieve the maximum number of iterations. Once the number of maximum iterations is achieved, the terminate iteration cycle is commenced through Steps 3 to 5.

## 3. Harmony Searching Algorithm Improvement

Compared with other optimization algorithms, the HS algorithm is superior on the basis of the following reasons. (1) The HS algorithm requires minimal mathematical criteria and does not entail variable initialization. (2) The entire search process of the HS algorithm assumes a completely random pattern without considerable manual intervention. (3) The HS algorithm considers the entire available acoustic memory information to create a new harmony vector. Given these advantages, the HS algorithm has been the focus of attention of many foreign scholars since 2001.

The randomness of the algorithm results in low precision. The HS algorithm is mainly adopted to improve the accuracy of the optimization problem. The HS algorithm is a strong randomness heuristic algorithm, has a simple structure, is easily operated, and involves only a few parameters and other characteristics. However, the HS algorithm also adopts sensitive parameters and generates slow convergence defects, thereby entailing further research on enhancements. The HS algorithm uses a few paramount parameters that directly affect the algorithm. These parameters include the harmony memory probability values PAR_max_ and PAR_min_ and the fine-tuning probability BW. In this study, the accuracy of the HS algorithms is improved to prevent attaining a premature local optimum. In this regard, the following enhancements were applied.

### 3.1. Construction of a New Harmony HM Database

In the original harmony algorithm, harmony memory acquisition is random, thereby entailing a relatively large stochastic algorithm. This effect reduces the accuracy of the algorithm. In this study, a rough set was employed on the upper and lower boundaries to establish a new harmony HM database. Rough set theory was adopted to reduce the randomness of the harmony memory database and improve the latter's accuracy.

**Step 1 .**

The relationship 1 ≤ *i* ≤ *k* was applied, where *k* is the clustering center, to establish the initial average *Z*_*i*_.

**Step 2 .**

With the data points *x*_*i*_, 1 ≤ *i* ≤ *n*, the limits of the upper and lower boundaries, $\bar{B}U_{i^{\prime }}$ and $\underline{B}U_{i^{\prime }}$, respectively, were almost reached. $\bar{B}U_{i^{\prime }}$ and $\underline{B}U_{i^{\prime }}$ are the limits of the clustering center *U*_*i*′_. *U*_*i*′_ was adopted to denote the distance between two points *d*_*i*′*j*_ − *d*_*ij*_.

**Step 3 .**

If *d*_*ij*_ corresponds to the extreme minimum value, then *d*_*i*′*j*_ must be close to *d*_*ij*_. If *d*_*i*′*j*_ − *d*_*ij*_ is less than a given threshold, then $x_{ij}\in \underline{B}U_{i^{\prime }}$; otherwise, $x_{ij}\in \bar{B}U_{i^{\prime }}$.

**Step 4 .**

A lower limit is established on the matrix, as follows:
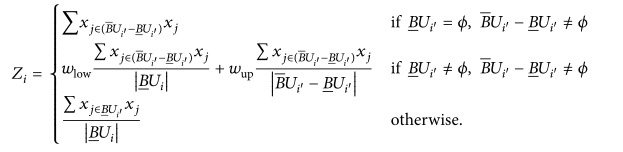
(3)

According to the previously presented steps, alternate data were used for preliminary screening to establish the harmony algorithm. However, more accurate data were required by the algorithm. Thus, the *K*-nearest neighbor (KNN) algorithm was employed to attain the appropriate harmony memory matrix. *K* denotes a given clustering center based on prior knowledge.

The KNN method [22] was originally proposed in 1968 by Cover and Hart. The KNN is a theoretically more mature classification algorithm. The core idea of the KNN algorithm is simple: if a sample feature vector space *k* most similar (the nearest feature vector space) to the sample belongs to the major category, then the sample likely belongs to such category. The KNN method for the decision-making category is based solely on the nearest category or several categories of samples to which samples are designated to. The traditional KNN algorithm has been referred to as an example-based learning classification algorithm. By comparing each training sample, users find the text to be classified with the most similar *K* text. Finally, the text that contains the greatest number of similar categories is selected and classified as category text. The related mathematical expression is as follows:

(4) $$\begin{array}{c} p\left(\;d_{i},C_{j}\;\right)=\underset{d_{j}\in KNN}{\sum }\mathrm{sim}\left(\;d_{i},d_{j}\;\right)y\left(\;d_{i},C_{j}\;\right), \end{array}$$

where *d*_*i*_ is the feature vector, sim(*d*_*i*_, *d*_*j*_) corresponds to similarity, and *y*(*d*_*i*_, *C*_*j*_) denotes the classification properties. If *d*_*i*_ belongs to *C*_*j*_, then the value of the function is 1; otherwise, the value is 0. Herein, we used this kind of thinking process for classification.

In distributing the matrix *Z*_*i*_ sample items across the class space, we applied Euclidean distance as a distribution rule as follows:

(5) $$\begin{array}{c} d\left(\;x_{i},x_{j}\;\right)=\sqrt{\overset{n}{\underset{k=1}{\sum }}{\left(v_{k}\left(x_{i}\right)-v_{k}\left(x_{i}\right)\right)}^{2}}. \end{array}$$

If *Z*_*i*_ = {*z*_1_, *z*_2_,…, *z*_*n*_}, then each area involves a *k* clustering center. The aforementioned methods were adopted to establish a suitable search memory database HM as follows:

(6) $$\begin{array}{c} HM=K_{1i}*K_{2i},\ldots ,K_{Ni}. \end{array}$$

**Step 5 .**

When all the optional data maximum and minimum values were less than the threshold *T*, the loop was terminated. Otherwise, Steps 2 to 4 were repeated to establish the appropriate database matrix of harmony. Herein, we considered *w*_up_ = 1 − *w*_low_,  0.5 < *w*_low_ < 1.

Matrix HM_*Z*_*i*__ was established by using the new harmony matrix of rough set theory. By using rough set theory to establish a harmony matrix principle instead of a random matrix, we avoided the poor robustness and randomness of the HS algorithm.

### 3.2. Probability PAR Adjustment

A study on the HS algorithm revealed that probability PAR tuning and volume BW are set randomly or by experience. In such case, no change in the convergence process is achieved. In fact, the effect of these two parameters on the convergence of the algorithm is relatively large, particularly in the latter part of the run. The original HS algorithm is not concerned with this aspect; it is not conducive for a fast algorithm that converges to the global optimum. In this study, the PAR and BW parameters in the original HS algorithm were improved to avoid falling into the local optimum.

In the HS algorithm, adjusting the probability PAR is also an important component. In the literature [10], the value of a small PAR has been shown to enhance the local search ability of the algorithm. By contrast, the value of a larger PAR is beneficial for adjusting the search area. The expression is shown as follows:

(7) $$\begin{array}{c} PAR_{T}=PAR_{min}+\frac{PAR_{max}-PAR_{min}}{T}*t, \end{array}$$

where *T* is the iteration number and *t* is the current number of iterations. In this study, the global search algorithm was improved by introducing a feedback mechanism and moving a step length. The number of iterations *T* was also updated. To update the probability of the harmony memory database and step length, we adopted the following expression:

(8) $$\begin{array}{c} T=\frac{\sum_{i=1}^{c}\left(X_{best}^{i}-X_{worst}^{i}\right)}{\sqrt{{\sum_{i=1}^{c}\left(X_{best}^{i}-X_{worst}^{i}\right)}^{2}}}. \end{array}$$

The *t* times moving steps were expressed as follows:

(9) BWT=BWmin+BWmax−BWmin∗TtTmaxif  T<Tmax2BWmaxif  T≥Tmax2.

By improving the HS algorithm using dynamic tone control, adjustable probability PAR values and bandwidth BW were attained, overcoming the shortcomings in probability PAR value and bandwidth generated by the fixed tone control in the basic HS algorithm. Compared with other algorithms, whether on test function or vector search solutions, the enhanced HS algorithm exhibited a better performance.

### 3.3. Termination Conditions

At the maximum or minimum harmony database values less than the threshold *T*, the loop was terminated. Otherwise, the original HS algorithm was repeated from Steps 2 to 4.

## 4. Fuzzy Clustering Segmentation

The FCM clustering algorithm was proposed using fuzzy set theory. The FCM uses fuzzy set theory for classification. Data under a certain degree of categorization is divided into various types, and cluster centers are calculated in accordance with all the updated data objects of each category. This ambiguity makes the classification process of the FCM algorithm better reflect the actual data distribution, particularly for the treatment of overlap between all categories.

FCM clustering image segmentation treats pixels in an image as a cluster sample and the entire diagram as a sample set; each pixel feature vector is extracted from the image and regarded as the sample; then the pixels in the feature space are clustered. In essence, the pixels with similar characteristics are grouped in an aggregate class, whereas the pixels with dissimilar features are distributed into different classes. Finally, each pixel is completely tagged to image segmentation.

In the fuzzy means clustering algorithm (FCM), the initial value setting *c* is a more important direct effect of segmentation speed, accuracy, and effectiveness. Before starting, the cluster number must be given first. However, in the absence of human intervention and prior knowledge of the image, such as in an automated system, determining the cluster number is a difficult task. Therefore, *c* values based on image segmentation problems are difficult to determine under fuzzy clustering. In traditional FCM, the initial value *c* is random. Thus, the randomness of the algorithm is high and a local optimal solution is attained.

In this study, the initial value is regarded as the number of optimal solutions obtained by the HS algorithm; the algorithm can achieve a favorable result by avoiding the local optimal solution. The MRI brain image segmentation effect attained by the improved algorithm is better than that achieved through the traditional FCM. A previous study [17] promoted the objective function of the FCM clustering algorithm; the related expression is as follows:

(10) $$\begin{array}{c} J\left(\;U\in M_{hc},V\;\right)=\overset{c}{\underset{j=1}{\sum }}\;\overset{N}{\underset{k=1}{\sum }}u_{ji}d_{ij}^{2}, \end{array}$$

where *U* = [*u*_*ki*_]_*c∗n*_ ∈ *M*_*hc*_, *m* is the fuzzy index, and *d*_*ij*_ is the distance between the clustering center and clustering objects. In this study, the Euclidean metric distance was adopted to compute the gray difference between any point and the cluster center. The Euclidean metric distance can be calculated with minimum steps.

The minimum value refers to the direction of clustering in ∑_*k*=1_^*c*^ *u*_*ki*_ = 1 under the condition of the constraint *J*_*m*_(*U*, *V*). By using the Lagrangian approximation solution, the degree of membership and cluster center under the extreme value are calculated as follows:

(11) JmU,V=∑i=1c ∑j=1Nuijmdji2+∑j=1Nλi∑i=1cuji−1,

(12) ujxi=1/xi−mj21/b−1∑k=1c1/xi−mj21/b−1,i=1,2,…,n,  j=1,2,…,c,

(13) vi=∑k=1Nukimxk∑k=1Nukim,i=1,2,…,c.

Equations (9) and (11) were utilized by continuous iterative optimized clustering. Each iteration was adopted to calculate the membership degree matrix and cluster center until convergence was reached.

The detailed steps for FCM calculation are as follows:

(1) The optimal value of *c* was obtained using the improved HS algorithm and rough set theory as the initial value *c* for the FCM algorithm.
(2) The clustering center vector *V* = [*v*_1_, *v*_2_,…, *v*_*c*_] was initialized.
(3) On the basis of (10), the membership degree matrix *U* ^(*t*)^ = [*u*_*ki*_^(*t*)^]_*c∗n*_ was updated, where *t* denotes the iteration number.
(4) Equation (11) was employed to update the clustering center *V*′ = [*v*_1_′, *v*_2_′,…, *v*_*c*_′].
(5) The number of iterations *T* or error parameter *ε* when *t* < *T* or |*V* − *V*′ | > *ε* was determined. Then, Steps 3 to 5 were repeated until the loop was terminated.

## 5. Experiment

In a simulation experiment, the improved harmonic search algorithm and the original harmony algorithm were used to obtain the optimal, worst, and average values for MRI brain images 1–4 (MRI1–4).

In the data index, all values of the improved HS algorithm were superior to those of the original HS algorithm. The improved HS algorithm also obtained a better optimal solution. The different values were computed using Euclidean distance. The quantitative units were expressed to 10^5^.

In the experimental data (Table 1), a smaller optimal value indicates a nearer distance to the clustering center and a more accurate selection of the cluster center. As the average value approaches the optimal value, the more optimal condition of the cluster center is achieved. More precise cluster centers attain better segmentation effects.

The coefficient segmentation function *V*_pc_ and entropy segmentation function *V*_pe_ were utilized to qualitative analyze the experimental results [23, 24]. The related expressions are as follows:

(14) Vpc=1n∑i=1c ∑j=1nuij2,Vpe=−1n∑i=1c ∑j=1nuijlog⁡uij.

In the simulation experiments, the improved search algorithm and fuzzy clustering segmentation were compared with the original fuzzy clustering (FCM) segmentation algorithm. The experimental results are shown in Table 2.

The value of the coefficient segmentation function *V*_pc_ of the improved algorithm was greater than that of the FCM algorithm (Table 2). Conversely, *V*_pe_ values were lower in the improved algorithm than in the FCM algorithm. For image segmentation, a high *V*_pc_ or a low *V*_pe_ indicates perfect segmentation effects.

Consequently, the segmentation effect of the improved algorithm is better than that of the FCM algorithm. The data of the FCM algorithm shown in Table 2 reveal that the values of *V*_pc_ and *V*_pe_ are closed. This result can be explained by the fuzzy clustering algorithm in images MRI1 and MRI2, which involved 20 and 23 iteration times into the local optimal solution, respectively. Moreover, in 21 and 23 iteration times, MRI3 and MRI4 fell into the local optimal solution. Thus, the existence of the local optimal solution rendered the FCM algorithm not ideal for MRI image segmentation.

By using the improved algorithm, MRI1–4 brain image segmentation obtained different initial values of *c*. The differences in initial value also changed the partitioning membership.

The final segmentation results for MRI1 and MRI2 are shown in Figure 2. When the improved algorithm was used to determine the partition *c* = 3, the original FCM algorithm was set to *c* = 3. Meanwhile, the final segmentation results for MRI3 and MRI4 are shown in Figure 3. When the improved algorithm was used to determine the partition *c* = 4, the original FCM algorithm was also set to *c* = 4.

**Figure 1:**
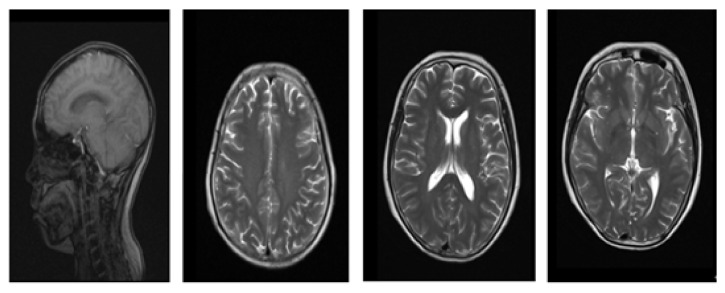
Original MRI images.

**Figure 2:**
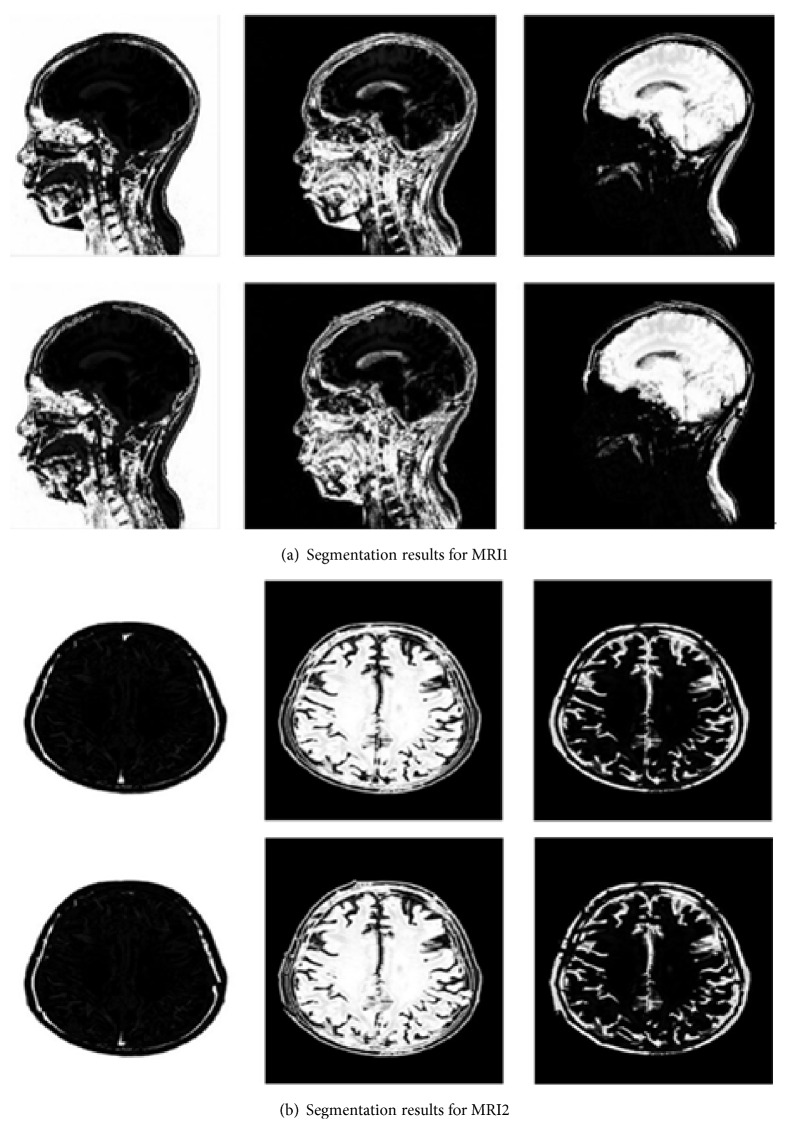
Segmentation results for the MRI1 and MRI2 brain images.

Figure 1 displays the experimental data for images MRI1–4. Meanwhile, Figure 2 shows the segmentation results for images MRI1 and MRI2. The membership is associated with the initial value *c* = 3. The first and second rows display the segmentation effects. The first row shows the experimental results for the improved HS algorithm and FCM. The second row shows the experimental results obtained using the original FCM algorithm. The segmentation results reveal that the fuzzy clustering method generated an oversegmentation phenomenon. For the actual MRI brain image segmentation effect, the algorithm proposed in this study performed better than the original FCM algorithm.

**Figure 3:**
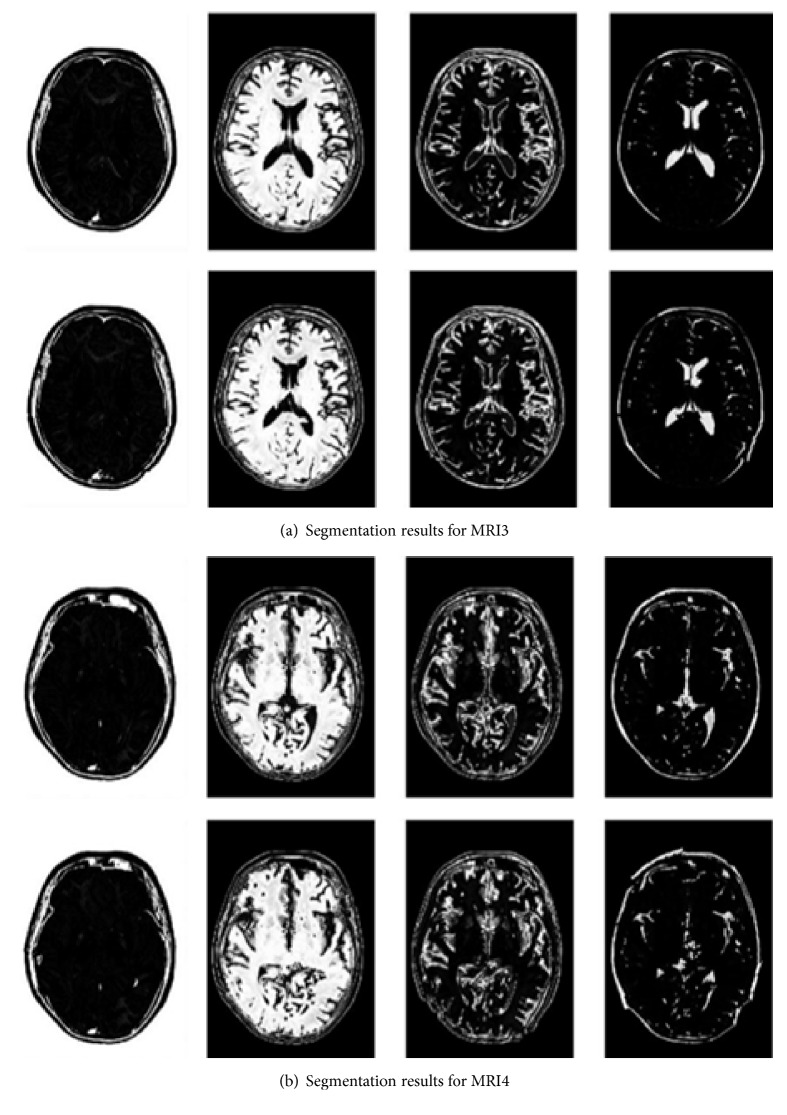
Segmentation results for MRI3 and MRI4.

In Figure 3, the segmentation effect was affected by membership; the membership degree was associated with the initial *c* = 4. The first row shows the experimental results of the improved HS algorithm and FCM. The second row shows the experimental results obtained using the original FCM algorithm. The segmentation results reveal that the proposed MRI brain image segmentation effect obtained using the improved algorithm is better than that of the fuzzy clustering algorithm. In the fuzzy clustering algorithm, the initial value of uncertainty generated a local optimum algorithm, which affected the segmentation.

## 6. Conclusion

In this study, MRI brain image segmentation was achieved using the HS algorithm and the fuzzy clustering algorithm. The HS algorithm is more extensively used. However, given its drawbacks, the algorithm easily falls into the local optima. Thus, this study proposed an improved HS algorithm for MRI brain segmentation. Rough set theory was adopted to achieve an improved HS algorithm of an optimal harmonic database and important probability parameters for promoting harmony contraction convergence. Then, brain images were segmented using the fuzzy clustering algorithm. The initial value in the fuzzy clustering algorithm was random, which affected the segmentation. Therefore, the optimal harmony value obtained by the improved HS algorithm was used as the initial value of the fuzzy clustering algorithm. The uncertainty in the initial value of the fuzzy clustering algorithm was avoided, thereby preventing the algorithm from falling into the local optimum. The simulation experiments showed that the proposed method produces better segmentation effects than those of the original fuzzy clustering algorithm.

## Figures and Tables

**Table 1:** Experimental results and comparison of data.

Image	Algorithm	Best value	Worst value	Average value
MRI1	Improved HS	2.17848	2.40321	2.18952
	HS	2.37648	3.17136	2.48956

MRI2	Improved HS	4.68368	5.03184	4.69925
	HS	4.78272	5.29190	4.89628

MRI3	Improved HS	4.24348	6.88194	4.25740
	HS	4.37389	9.16356	5.06598

MRI4	Improved HS	3.35994	4.70662	3.45236
	HS	4.5226	5.00091	4.63587

**Table 2:** Experimental results and comparison of data.

Image	Algorithm	*V*_pc_	*V*_pe_	Iterations
MRI1	Improved algorithm	0.873789	0.231145	27
	FCM	0.689989	0.656345	20

MRI2	Improved algorithm	0.881977	0.314585	29
	FCM	0.699575	0.642841	23

MRI3	Improved algorithm	0.903779	0.217534	29
	FCM	0.668798	0.656146	21

MRI4	Improved algorithm	0.887907	0.334695	29
	FCM	0.657174	0.632898	23
